# Anticholinergic Burden in Individuals with Down Syndrome: An Examination of Risk and Clinical Implications

**DOI:** 10.3390/jpm16080398

**Published:** 2026-07-26

**Authors:** Emanuele Rocco Villani, Rosa Liperoti, Laura Franza, Francesca Maria Secciani, Antonella Di Paola, Graziano Onder, Angelo Carfì

**Affiliations:** 1 Dipartimento dell’ Integrazione, Unità Operativa Complessa Geriatria Territoriale, Azienda Unità Sanitaria Locale Modena, 41122 Modena, Italy; e.villani@ausl.mo.it; 2 Department of Geriatric, Università Cattolica del Sacro Cuore, 00168 Rome, Italy; rosa.liperoti@unicatt.it; 3 Department of Geriatric, Fondazione Policlinico Universitario A. Gemelli, Istituto di Ricerca e Cura a Carattere Scientifico, 00168 Rome, Italy; graziano.onder@unicatt.it; 4 Clinical Experimental Medicine, Università degli Studi di Modena e Reggio Emilia, 41122 Modena, Italy; 5 Dipartimento di Emergenza-Urgenza, Azienda Ospedaliero-Universitaria, 41125 Modena, Italy; 6 Scuola di Specializzazione in Pediatria, Alma Mater Studiorum—Università di Bologna, 40126 Bologna, Italy; 7 Department of Geriatrics, Centro Medicina dell’ Invecchiamento, Fondazione Policlinico Universitario A. Gemelli, Istituto di Ricerca e Cura a Carattere Scientifico, 00168 Rome, Italyangelo.carfi@policlinicogemelli.it (A.C.)

**Keywords:** Down syndrome, anticholinergic burden, personalized medical approach, polypharmacy, deprescribing, multimorbidity

## Abstract

**Background/objective**: Down syndrome (DS) is characterized by premature aging, with comorbidities observed in the older population without DS, a high prevalence of multimorbidity and cognitive decline. The use of multiple medications further increases clinical complexity. The objective of this paper is to evaluate anticholinergic use and the anticholinergic burden (ACB) in a sample of persons with DS. **Methods**: cross-sectional retrospective study involving community-dwelling persons with DS, afferent to the DS clinics of the Fondazione Policlinico Universitario “A. Gemelli” (Rome, Italy). Individuals were assessed through a standardized clinical protocol. ACB was evaluated according to the anticholinergic cognitive burden scale, with a score ≥ 3 indicating high risk of anticholinergic side effects. **Results**: 337 individuals were taking ≥ 1 chronic medication, mean age was 37.7 (±14.6), 158 (43.8%) were females. 143 (39.6%) individuals took ≥ 1 medication with known anticholinergic burden, 51 (14.1%) individuals showed high risk ACB score. High risk ACB score prevalence increased with age (<18 years *n* = 0, 0%; 18–39 years *n* = 14, 8.2%; 40+ years *n* = 37, 22.3% *p* < 0.001). Polypharmacy was more prevalent among individuals with high ACB score (*n* = 20, 39.2% vs. *n* = 23, 7.4%, *p* < 0.001). The most prevalent drugs in individuals with high risk ACB score were antipsychotics (*n* = 34, 66.6% vs. *n* = 17, 33.3%, *p* < 0.001). The most prevalent comorbid condition was psychosis (*n* = 34, 66.6% vs. *n* = 16, 33.3%, *p* < 0.001). **Discussion**: individuals with DS show a high prevalence of anticholinergic drug use, increasing with age and associated with polypharmacy and psychiatric conditions. These medications are directly addressing the underlying psychiatric or neurological conditions in DS, rather than causing them. Nevertheless, these medications are often essential for managing psychiatric disorders, simultaneously increasing ACB, posing a risk of exacerbating pre-existing vulnerabilities (e.g., cognitive decline), which warrants the need for a more personalized strategy. **Conclusions**: A thorough assessment of drug history is mandatory in the DS population, to apply deprescribing, pharmacological switches, non-pharmacological approaches, to lower or even prevent high ACB, promoting overall a personalized and tailored approach to this population.

## 1. Introduction

Down syndrome (DS) is a genetic condition defined by the presence of an extra copy of chromosome 21. This trisomy results in a unique phenotype that includes characteristic physical features, intellectual disability, and a range of associated health conditions [1]. One of the most striking features of DS is its association with premature aging, which makes individuals with DS more susceptible to age-related comorbidities commonly observed in the general elderly population: they are increasingly prone to chronic conditions such as hypothyroidism, cardiovascular disease, and dementia, further complicating their clinical profile and often requiring complex medication regimens [2]. The elevated burden of multimorbidity in DS frequently leads to polypharmacy, i.e., the use of multiple medications to manage symptoms and prevent disease progression [3]. However, complex pharmacological management also increases the likelihood of adverse drug interactions and side effects, especially when medications with anticholinergic properties are prescribed. Anticholinergic medications block acetylcholine, a neurotransmitter critical for memory, cognition, and autonomic bodily functions. In the general population, high anticholinergic burden (ACB) is linked with cognitive decline, increased risk of falls, and overall functional decline [4]. For people with DS, who already experience cognitive challenges, the impact of anticholinergic drugs could be particularly detrimental.

Recent studies have shown that anticholinergic burden not only correlates with cognitive impairment but also exacerbates symptoms of existing conditions like dementia, a common age-related comorbidity, defined as those diseases concomitantly present with DS [5]. To quantify anticholinergic burden, the Anticholinergic Burden (ACB) scale is commonly used; this scale classifies medications according to their anticholinergic impact, with a score of 3 or higher considered high risk for adverse cognitive effects [6]. Although there is growing concern about the use of anticholinergic medications in older adults, data on their impact on people with DS is sparse, despite the fact that this group often experiences accelerated aging and related complications [7].

This study aims to fill this gap by evaluating the prevalence of anticholinergic use and associated with high ACB scores among individuals with DS. Specifically, it examines associations between anticholinergic burden, age, gender, comorbidities, and polypharmacy, providing critical insights for clinicians who manage the care of individuals with DS.

## 2. Materials and Methods

This is an observational, cross-sectional, single-center study. All participants were individuals with DS enrolled from those attending the geriatrics outpatient clinics of the Fondazione Policlinico Universitario “Agostino Gemelli” IRCCS, Università Cattolica del Sacro Cuore in Rome, Italy. Data were collected from 2012 to 2021. No specific sample calculation was performed, as the population of interest is inherently small. No specific exclusion criteria were applied, except for those who declined participation. Participants were referred to our clinics by DS associations, and family physicians.

The evaluations followed a standardized clinical protocol that included a physical examination, electrocardiogram, blood sampling, physical performance assessment, and a nutritional assessment. Depending on individual clinical needs, additional assessments were conducted, e.g., neuropsychological evaluation, echocardiography, dual-energy X-Ray absorptiometry scanning, hearing evaluation by an otolaryngologist, pulmonary assessment for obstructive sleep apnea syndrome, and gastroenterological assessment for celiac disease. Chronic conditions were identified based on clinical history, comprehensive physical examination, and review of medical records [8].

Clinical and medical history data for all patients were gathered using the Information System of the Policlinico Universitario Agostino Gemelli IRCCS. Diseases were coded according to the International Classification of Diseases-10 (ICD-10) [9], and all medications patients were taking as part of their chronic treatment regimen on the day of their visit were recorded by study physicians and coded according to the Anatomical Therapeutic Chemical (ATC) Classification. Over-the-counter medications were not included. Multimorbidity was defined as the presence of two or more chronic diseases [10], while polypharmacy was defined as the concurrent use of five or more medications [11]. Diagnoses were grouped by clinical significance, and medications were categorized according to ATC level 4. The ACB score was calculated for each individual, with scores of 3 or higher indicating a high risk of adverse anticholinergic side effects [6].

Data are reported as relative frequencies for categorical variables and mean ± standard deviation (SD) for continuous variables. Normality was assessed using the Shapiro–Wilk test and visual inspection of Q-Q plots. Continuous variables not following a normal distribution were reported as median and interquartile range (IQR). The sample was also stratified by age group (ages 18–39, and over 40 years). These age groups were chosen to account for the higher prevalence of chronic conditions observed in DS after age 40 [3]. Intellectual disability (ID) was omnipresent in the sample and, therefore, was not included in the multimorbidity count. Between-group differences were analyzed using independent samples t-tests for normal continuous variables, Mann–Whitney for non-normal continuous variables and chi-square tests for categorical variables. A multivariable logistic regression analysis was run, taking into consideration variables with clinical relevance and previous evidence regarding factors potentially associated with anticholinergic burden. Age and sex were included as fundamental demographic variables [12,13,14]. Selected comorbidities (psychosis, dementia, depression, autism spectrum disorders, epilepsy, and osteoporosis) were entered into the model because they showed significant between-group differences and were considered clinically plausible determinants of anticholinergic burden in adults with DS [15]. A conceptual directed acyclic graph (DAG) was then developed to summarize the hypothesized relationships between demographic characteristics, multimorbidity, psychiatric comorbidities, polypharmacy, and ACB. The DAG was used to support the identification of clinically relevant variables for inclusion in the multivariable model. Multicollinearity was assessed through variance inflation factors (VIF), and model fit was evaluated using the Hosmer-Lemeshow goodness-of-fit test and Nagelkerke’s pseudo-R^2^. Bonferroni and Holm correction were both used to correct for multiple testing. Statistical significance was set at *p* < 0.05. All analyses were performed using R version 4.4.1 (14 June 2024 ucrt, we used the ggplot2 [16]), dyplr [17], tidyverse [18], and officer [19] packages.

## 3. Results

Table 1 shows the sample characteristics according to ACB. In the study cohort, the mean age was 38.4 years (SD 12.8), with a slight male predominance. Approximately 39.6% of participants were prescribed at least one medication with known anticholinergproperties. Importantly, 15.1% of the participants scored ≥3 on the ACB scale, placing them at high risk for adverse cognitive and physical side effects due to anticholinergic medication use.

**Figure 1:**
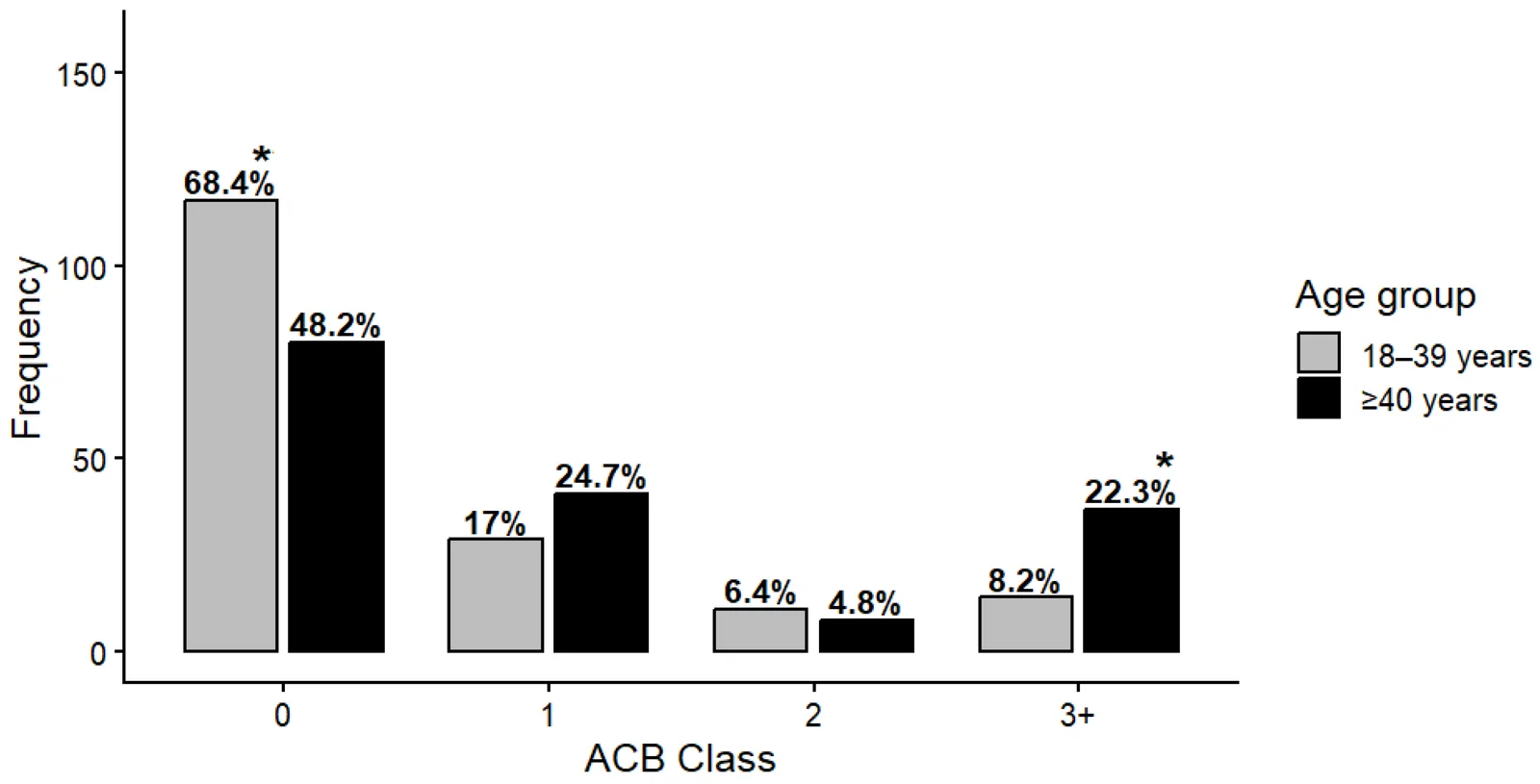
Anticholinergic burden (ACB) by age group. * indicates *p*-value is <0.001.

The analysis revealed no statistically significant difference in high-risk ACB scores between males and females, suggesting that both genders may be equally vulnerable to anticholinergic burden in the context of DS. However, age played a significant role in anticholinergic burden: 8.2% of those aged 18–39 and 22.3% of those aged 40 or older fell into the high-risk category (*p* < 0.001). These results are shown in Table 2 and are depicted in Figure 1.

All individuals with high ACB scores had multimorbidity, and its prevalence was very high among those with low ACB scores (>90%), not allowing us to adjust the multivariable analysis for this variable. Polypharmacy was substantially more common among those with high ACB scores, with 39.2% of these individuals taking five or more medications, compared to only 7.4% of those with lower ACB scores (*p* < 0.001). We observed that the most common type of medication contributing to high ACB scores was antipsychotics, used by 66.6% of high-risk individuals. This usage was strongly associated with a diagnosis of psychosis, which was present in two-thirds of those with high ACB scores (*p* < 0.001). The medications taken by patients are described in detail in Table 3.

Additionally, other drug classes, including antidepressants, anxiolytics, and antiepileptics, were more frequently prescribed to individuals with high-risk ACB scores, reflecting patterns of complex psychiatric and neurological comorbidities in this group. These medications, while essential for managing mental health and seizure disorders, contribute significantly to the overall anticholinergic load, potentially exacerbating cognitive decline and functional impairments.

In the multivariable model (Table 4), age and polypharmacy were strongly associated with ACB, with polypharmacy showing the strongest association, supporting the central role of cumulative medication exposure. Specific comorbidity patterns—particularly psychosis, depression, autism spectrum disorders, and osteoporosis—were associated with elevated ACB, also after correcting for age and sex. In contrast, sex, dementia, and epilepsy were not associated with high ACB.

## 4. Discussion

The findings of this study highlight the considerable anticholinergic burden faced by individuals with DS, particularly those who are older and have multiple chronic conditions.

The comparison between the prevalence of a high anticholinergic burden (ACB ≥ 3) in adults with Down syndrome (DS) and that in the general population, including elderly individuals, highlights notable differences that underscore the unique health challenges faced by individuals with DS. In our study, 14.1% of participants with DS had an ACB score ≥ 3, a surprisingly high percentage, considering that this cohort also includes young adults. While the prevalence of high ACB does increase with age in individuals with DS, the rate observed in our study remains on average higher than in the general adult population, in which high ACB scores are more commonly seen among older adults—typically individuals aged 65 and above [20].

In the general older adult population, ACB ≥ 3 is reported in approximately 20–25% of individuals aged 65 and over, reflecting the increase in polypharmacy and chronic conditions in older age [21]. However, unlike the general population, where high anticholinergic burden is primarily associated with advanced age and its neuropsychiatric-associated conditions [22], individuals with DS are at an elevated risk of high ACB even at younger ages, due to the frequent use of multiple medications, particularly those with anticholinergic properties such as antipsychotics, antidepressants, and antiepileptics [23]. ACB often arises from medications prescribed for non-neurological or non-psychiatric conditions (e.g., COPD, asthma, urinary incontinence) [24]. These medications inadvertently contribute to anticholinergic effects, which may trigger or worsen neurological or psychiatric symptoms, such as cognitive decline, dementia, or depression. For individuals with DS, the scenario appears partly reversed: the high prevalence of psychiatric conditions in DS, such as psychosis, depression, or epilepsy, necessitates treatment with medications like antipsychotics, antidepressants, and antiepileptics [25].

Our study shows that these are the actual drug classes most responsible for high ACB in the DS population (e.g., antipsychotics accounted for 66.6% of high ACB cases in the study). The challenge is that while these medications are often essential for managing psychiatric disorders, they simultaneously increase ACB, posing a risk of triggering or exacerbating pre-existing vulnerabilities. In our study, ACB in individuals with DS is frequently observed in the context of psychiatric disorders requiring psychotropic treatment, suggesting it may not be solely an unintended consequence of prescribing for non-psychiatric conditions [26]. This makes addressing the burden even more critical, as it directly ties into the patient’s quality of life and cognitive function. Given the known risks associated with anticholinergic medications—particularly cognitive impairment, there is an urgent need for careful medication management in this population. The high prevalence of psychotropic medications like antipsychotics and antidepressants among individuals with high ACB scores suggests that there is a challenge of treating psychiatric comorbidities in DS without exacerbating anticholinergic load needing to be addressed [27]. Given the association between ACB and multimorbidity, it is essential for clinicians to consider alternative treatments with lower anticholinergic profiles wherever possible. Our data does indeed show an exceptionally high prevalence of multimorbidity in the study cohort, with over 95% of participants experiencing two or more chronic conditions [28]. While this finding reflects the accelerated aging seen in DS, the high prevalence of multimorbidity could flatten its clinical implications [29].

Interestingly, our analysis found only few significant differences in the prevalence of comorbidity between individuals with high ACB scores (ACB ≥ 3) across different age groups, which were less significant than the ones observed in individuals with lower ACB scores (ACB < 3). This suggests that once DS adults have a high ACB score, the prevalence of comorbidities remains consistently high, regardless of whether they are under or over 40 years old [30]. This consistency across age groups among those with ACB ≥ 3 suggests that high anticholinergic burden—often associated with polypharmacy and conditions requiring multiple medications—might be a more pivotal factor in the overall health status of DS adults than chronological age alone [27]. However, among DS adults with an ACB score of less than 3 (ACB < 3), we observed notable differences in comorbidity prevalence when comparing the younger (18–39 years) and older (40+ years) age groups. Older adults with DS with lower ACB scores had a higher prevalence of certain comorbid conditions, supporting the notion that age itself remains a significant risk factor for multimorbidity in DS individuals when anticholinergic burden is minimized. This age-related increase in comorbidities aligns with trends observed in the general population, where advancing age is commonly associated with an elevated risk of chronic diseases [31].

Among comorbidities, patients presenting with psychosis and autism spectrum disorders were more likely to present with high anticholinergic burden, followed by osteoporosis and depression. In contrast, dementia and epilepsy did not appear to be associated with high ACB. These findings suggest that in individuals with DS, high ACB could be driven by specific comorbidity patterns rather than by neurological comorbidities per se. The association between osteoporosis and high anticholinergic burden may appear unexpected; however, in DS, reduced bone mineral density is common from early adulthood and shows a premature, progressive course [32]. Cognitive decline itself has been linked to bone loss independently of age [33], while antipsychotic use—one of the main contributors to ACB in this population—is also associated with reduced bone density [34]. Taken together, these findings suggest that osteoporosis may represent a core feature of DS that is further exacerbated by psychotropic treatment patterns, contributing to higher ACB.

The exploratory nature of this study and the number of statistical comparisons performed introduce the possibility of type I error. Post hoc corrections for multiple testing showed that the main associations identified in the multivariable model remained statistically significant, with the exception of depression. Yet, individual *p*-values should be interpreted with caution, and confirmation in independent cohorts will be necessary to establish the robustness and reproducibility of these findings.

### The Therapeutic Dilemma

The management of psychiatric and behavioral comorbidities in people with DS exemplifies a major therapeutic dilemma. Psychiatric disorders, including psychosis, depression, anxiety, and behavioral disturbances—are frequent in this population and often require pharmacological treatment to preserve patient safety and quality of life. Yet many of the most effective medications carry a moderate-to-high anticholinergic burden, raising the risk of cognitive deterioration, delirium, and accelerated functional decline [35]. The challenge is particularly acute given the already elevated vulnerability of individuals with DS to early-onset Alzheimer’s disease and dementia [36]. In such scenario, there could be an unavoidable trade-off: preserving psychiatric stability versus safeguarding cognition. Thereby, a more comprehensive and personalized approach should be evaluated to implement strategies that can help mitigate anticholinergic risk while maintaining psychiatric control. For instance, when alternatives exist, selecting drugs with minimal anticholinergic activity is critical. In psychiatry, this may mean preferring agents such as lurasidone, ziprasidone, brexpiprazole, and lumateperone or sertraline over clozapine or paroxetine. Such switches, when clinically feasible, can meaningfully reduce ACB without compromising efficacy and could be useful among people with intellectual disabilities [37].

Also, for patients requiring high-burden drugs, the lowest effective dose should be maintained, with periodic re-evaluation of necessity. Multidisciplinary medication reviews, often pharmacist-led, have been shown to safely reduce anticholinergic exposure in older adults and are applicable to DS. Deprescribing should be gradual and closely monitored, as improvements in cognition, mood, and daily function are possible when ACB is reduced [38]. The systematic use of validated tools such as the ACB scale can help clinicians quantify risk and identify high-priority cases for intervention. Regular cognitive assessments should be integrated into routine DS care [39].

It is also worth noting that symptoms may fluctuate and vary over time, allowing for cautious dose reduction or discontinuation [40]: behavioral and psychosocial interventions—including environmental adjustments, caregiver education, occupational therapy, and cognitive stimulation—can reduce psychotropics need. These measures do not replace medication in severe cases but may decrease the need for higher-risk pharmacological strategies [41].

It is worth noting that ACB associated with psychotropic medications is not uniform across drug classes or individual agents. Antipsychotics, antidepressants, and antiepileptic drugs differ substantially in their affinity for muscarinic receptor subtypes (M1, M2, and M3), as well as in their ability to cross the blood–brain barrier and exert central versus peripheral anticholinergic effects. In other words, two medications with the same ACB score may not necessarily produce equivalent clinical effects [42,43]. Antipsychotics such as clozapine and olanzapine, for instance, exhibit relatively high muscarinic receptor affinity, while lurasidone, ziprasidone, or aripiprazole have negligible direct anticholinergic activity [44]. Paroxetine, an antidepressant, is recognized as having substantial anticholinergic properties [45], while serotonin reuptake inhibitors such as sertraline or escitalopram show minimal muscarinic receptor antagonism [46]. The ACB scale was designed to provide a pragmatic estimate of cumulative anticholinergic exposure in routine clinical practice and not to capture these pharmacodynamic nuances [47]. Our findings reflect this limitation, and identify broad therapeutic categories associated with increased anticholinergic burden, and should not be interpreted as evidence that all medications within a given class carry comparable anticholinergic risk. Future studies incorporating drug-specific receptor profiles, dosage information, treatment duration, and pharmacogenetic determinants of drug metabolism may provide a more refined understanding of the mechanisms linking psychotropic prescribing and anticholinergic burden in adults with DS.

Another aspect that needs to be taken into account in this population is that medications may be differently metabolized, compared to the general population. There are different reasons behind this finding: for instance some drug-metabolizing enzymes, such as carbonyl reductase 1, are present on chromosome 21, which accounts for potential differences [48]. Also, it has been observed that people with DS can present with polymorphisms in the *CYP17* and *CYP19* genes in Cytochrome P450 [49], which is critical in processing many drugs, including ones directly impacting ACB [50].

**Figure 2:**
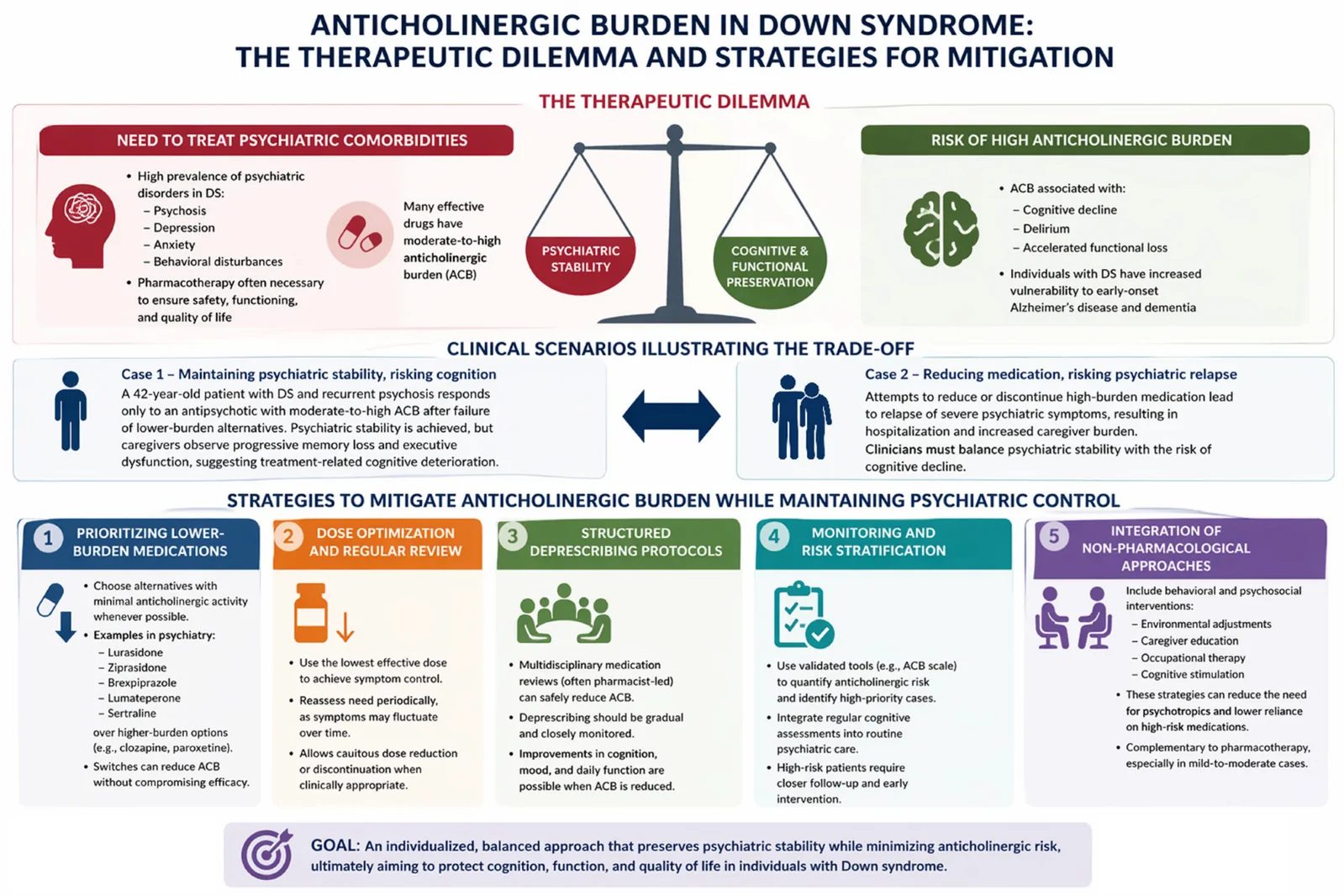
Physicians caring for people with DS have to keep in mind that, while treatment of psychiatric conditions is often necessary, it carries the risk of worsening both cognitive and physical performance. A careful balance between different aspects of patients’ and caregivers’ needs, through a personalized medicine approach, can allow minimize anticholinergic burden.

Overall, taking care of this group of patients requires a tailored and personalized medical approach, which takes into consideration the well-being of the patient, their needs and their expectations, similar to those needed for geriatric care [51]. The complexities of the therapeutic dilemma are summarized in Figure 2.

This study presents some limitations: in particular, its cross-sectional design does not allow us to have a follow up on the population and no cognitive tests were performed. Having used the ACB scale also presents some limiting aspects: indeed, even though it is widely used, it necessarily overlooks aspects regarding patients’ therapy, which could have an impact on the anticholinergic burden experienced by patients [52]. Also, ACB, in this context, acts as warning for potential future cognitive outcomes, not as an indicator of actual impairment.

Unfortunately, we did not have direct pharmacological and pharmacogenetic information, which is becoming more and more relevant, particularly in the context of adults with DS, thus we could only discuss existing literature. Yet, this study underscores the unique therapeutic dilemma in the management of individuals with DS: psychiatric comorbidities are highly prevalent and often require psychotropic medications that substantially contribute to ACB, creating a situation which needs to be navigated with extreme care by physicians and caregivers.

Finally, since the study population belongs to an outpatient clinic specialized in evaluating adults with DS, it may present a greater clinical complexity than the broader DS population. Thus, it could be argued that a selection bias could arise, particularly when considering the high multimorbidity burden. Yet, the prevalence of multimorbidity we observed in our cohort is comparable to that reported in other studies [53,54]. Also, this group of people is unlikely to be evaluated at a community level, as it presents a unique set of challenges and needs, which are likely to determine a referral to specialized clinics.

## 5. Conclusions

Unlike in the general population, where anticholinergic exposure frequently arises from treatment of somatic conditions, in DS it is intrinsically linked to the management of core psychiatric and neurological disorders. Our findings suggest that high ACB is frequently observed among individuals with psychiatric and neurological disorders requiring psychotropic treatment. This association highlights the need for individualized prescribing strategies aimed at balancing symptom control with minimization of anticholinergic burden.

From a personalized medicine perspective, anticholinergic burden in people with DS should be evaluated on individual multimorbidity profiles, psychiatric phenotypes, prescribing patterns while also considering chronological age. It is also worth noting that in this populations, differences in pharmacodynamic and pharmacokinetic responses have been reported and the cholinergic system itself appears intrinsically vulnerable [55]. Future pharmacogenetic markers should be integrated to identify those at greatest risk of anticholinergic adverse effects to guide individualized medication optimization strategies.

Mitigation strategies must therefore go beyond simple deprescribing and involve personalized approaches. These include preferential use of lower-burden agents, careful dose adjustments, structured medication reviews, and the integration of behavioral or psychosocial interventions that may reduce pharmacotherapy. Clinicians should regard anticholinergic burden reduction as a central aspect of care planning in this population, aiming to optimize psychiatric outcomes while minimizing the risk of accelerated cognitive decline.

## Figures and Tables

**Table 1:** Demographic and clinical sample characteristics. Bold indicates statistical significance.

Variable	Total (*n* = 337)	ACB < 3 (*n* = 286)	ACB ≥ 3 (*n* = 51; 15.1%)	*p*-Value
Age (mean, SD)	38.4, 12.8	38.9, 12.4	45.3, 11.6	**<0.001**
Female sex (*n*, %)	150, 44.5%	124, 43.4%	26, 51.0%	0.313
AGE > 40 (*n*, %)	166, 49.3%	129, 45.1%	37, 72.6%	**<0.001**
ACB (median, IQR)	0, 1	0, 1	4, 1.5	**<0.001**
Number of drugs (median, IQR)	2, 3	2, 2	4, 2	**<0.001**
Polypharmacy (*n*, %)	43, 12.8%	23, 8.0%	20, 39.2%	**<0.001**
Number of comorbidities (median, IQR)	9, 4	9, 3	10, 4.5	**<0.001**
Multimorbidity (*n*, %)	322, 95.6%	271, 94.8%	51, 100%	0.259
Comorbidities				
(*n*, %)				
Autism Spectrum Disorders	25, 7.4%	17, 5.9%	8, 15.7%	**0.032**
Psychosis	104, 30.9%	70, 24.5%	34, 66.7%	**<0.001**
Epilepsy	31, 9.2%	20, 7.0%	11, 21.6%	**0.002**
Dementia	73, 21.7%	56, 19.6%	17, 33.3%	**0.016**
Depression	45, 13.35%	34, 11.9%	11, 21.6%	**0.034**
Congenital Cardiopathy	105, 31.2%	95, 33.2%	10, 19.6%	0.078
Obesity	74, 21.0%	64, 22.4%	10, 19.6%	0.797
Visual Impairment	290, 86.1%	246, 86.0%	44, 86.3%	0.987
Hypoacusia	121, 35.9%	101, 35.3%	20, 39.2%	0.707
Thyroid Diseases	199, 59.1%	169, 59.1%	30, 58.8%	0.857
Celiac Disease	23, 6.8%	20, 7.0%	3, 5.9%	0.785
Obstructive Sleep Apnea	49, 14.5%	41, 14.3%	8, 15.7%	0.971
Osteoporosis	68, 20.2%	48, 16.8%	20, 39.2%	**<0.001**

**Table 2:** Age-related characteristics by anticholinergic burden. Bold indicates statistical significance.

Variables	ACB ≥ 3			ACB < 3		
	Age 18–39 Years (*n* = 14, 27.5%)	Age ≥ 40 Years (*n* = 37, 72.5%)	*p*-Value	Age 18–39 Years (*n* = 14, 27.5%)	Age ≥ 40 Years (*n* = 37, 72.5%)	*p*-Value
Females (*n*, %)	6, 42.9%	20, 54.1%	0.475	69, 43.9%	55, 42.6%	0.824
Multimorbidity (*n*, %)	14, 100%	37, 100%	NA	152, 96.8%	124, 96.1%	0.751
Polypharmacy (*n*, %)	3, 21.4%	17, 45.9%	0.110	8, 5.1%	15, 11.6%	**0.043**
Drugs by ATC (*n*, %)						
Antiepileptics	6, 42.3%	21, 56.8%	0.608	15, 9.6%	30, 63.8%	**0.002**
Antidepressants	8, 57.1%	19, 51.3%	0.911	25, 15.9%	31, 24.0%	0.086
Anxiolytics	1, 7.1%	6, 16.2%	0.062	2, 1.3%	2, 1.6%	0.843
Hypnotic/Sedatives	0, 0%	3, 8.1%	0.552	9, 5.7%	8, 6.2%	0.867
Antipsychotics	8, 57.1%	26, 70.3%	0.095	15, 9.6%	15, 11.6%	0.569
Antihistamines	1, 7.1%	0, 0%	0.275	4, 2.5%	4, 3.1%	0.778
Beta-blockers	2, 14.3%	0, 0%	0.071	2, 1.3%	2, 1.6%	0.998
Lipid-modifying drugs	1, 7.1%	1, 2.7%	0.695	4, 2.5%	9, 7.0%	**0.039**
Antiplatelets	1, 7.1%	0, 0%	0.275	0, 0.0%	4, 3.1%	**0.040**
Comorbidity (*n*, %)						
Psychosis	7, 50.0%	27, 73.0%	0.120	29, 18.5%	41, 31.8%	**0.009**
Dementia	0, 0%	16, 43.2%	**0.019**	0, 0%	52, 40.3%	**<0.001**
Depression	5, 35.7%	6, 16.2%	0.131	16, 10.2%	18, 14.0%	0.328
Epilepsy	3, 27.3%	8, 21.6%	0.988	4, 2.5%	16, 12.0%	**0.001**
Autism Spectrum Disorders	6, 42.9%	2, 5.4%	**0.003**	15, 9.6%	2, 1.6%	**0.005**
Congenital Cardiopathy	6, 42.9%	4, 10.8%	**0.010**	71, 45.2%	24, 18.6%	**<0.001**
Thyroid Diseases	10, 71.4%	20, 54.1%	0.261	96, 61.1%	73, 56.6%	0.435
Osteoporosis	4, 28.6%	16, 43.2%	0.524	25, 15.9%	23, 17.8%	0.668

**Table 3:** List of drugs in the sample. Bold indicates statistical significance.

Variable	Total (*n* = 337)	ACB < 3 (*n* = 286)	ACB ≥ 3 (*n* = 51)	*p*-Value
Thyroid Therapy	181 (53.71%)	157 (54.9%)	24 (47.06%)	0.378
Vitamin D supplement	137 (40.65%)	119 (41.61%)	18 (35.29%)	0.491
Antiepileptics	72 (21.36%)	45 (15.73%)	27 (52.94%)	**<0.001**
Antidepressants	83 (24.63%)	56 (19.58%)	27 (52.94%)	**<0.001**
Anxiolytics	11 (3.26%)	4 (1.4%)	7 (13.73%)	**<0.001**
Hypnotics/Sedatives	20 (5.93%)	17 (5.94%)	3 (5.88%)	0.892
Antipsychotics	64 (18.99%)	30 (10.49%)	34 (66.67%)	**<0.001**
Anti Dementia	1 (0.3%)	1 (0.35%)	0 (0%)	0.990
Antigout	28 (8.31%)	22 (7.69%)	6 (11.76%)	0.487
Bone Drugs	11 (3.26%)	7 (2.45%)	4 (7.84%)	0.116
Gastrointestinal Drugs	97 (28.78%)	75 (26.22%)	22 (43.14%)	**0.022**
ACE inhibitors	5 (1.48%)	4 (1.4%)	1 (1.96%)	0.854
Angiotensin 2 receptor Antagonists	5 (1.48%)	4 (1.4%)	1 (1.96%)	0.854
Cardiac Stimulants	10 (2.97%)	9 (3.15%)	1 (1.96%)	0.990
Lipid Modifying Drugs	15 (4.45%)	13 (4.55%)	2 (3.92%)	0.912
Diuretics	6 (1.78%)	4 (1.4%)	2 (3.92%)	0.496
Beta Blockers	6 (1.78%)	4 (1.4%)	2 (3.92%)	0.496
Glucose Lowering agents	14 (4.15%)	12 (4.2%)	2 (3.92%)	0.903
Anti Arrhythmics	1 (0.3%)	1 (0.35%)	0 (0%)	0.990
Antiplatelets	5 (1.48%)	4 (1.4%)	1 (1.96%)	0.975
Antihypertensives	7 (2.08%)	5 (1.75%)	2 (3.92%)	0.639
Antihistamines	9 (2.67%)	8 (2.8%)	1 (1.96%)	0.894
Pain Drugs	17 (5.04%)	14 (4.9%)	3 (5.88%)	0.758

**Table 4:** Logistic regression analysis.

Variable	aOR	95% CI	Raw *p*-Value	Holm-Adjusted *p*-Value	Bonferroni Adjusted *p*-Value	VIF
Age	**1.04**	**1.01–1.07**	0.071	0.284	0.710	1.58
Female sex	1.49	0.72–3.09	0.275	0.826	1.000	1.08
Polypharmacy	**4.31**	**1.86–9.95**	**<0.001**	**0.004**	**0.007**	1.12
Comorbidities						
Psychosis	**4.70**	**2.24–9.88**	**<0.001**	**<0.001**	**<0.001**	1.10
Dementia	1.06	0.43–2.59	0.794	0.826	1.000	1.36
Depression	**2.87**	**1.19–6.97**	**0.015**	0.073	0.147	1.08
Autism spectrum disorders	**5.45**	**1.61–18.39**	**<0.001**	**0.004**	**0.005**	1.27
Epilepsy	1.71	0.61–4.77	0.351	0.826	1.000	1.13
Osteoporosis	**3.92**	**1.73–8.88**	**<0.001**	**0.004**	**0.005**	1.17

Bold indicates statistical significance. CI: confidence interval, aOR: adjusted odds ratio. The multivariable model showed adequate calibration according to the Hosmer–Lemeshow goodness-of-fit test (χ^2^ = 7.54, df = 8, *p* = 0.480), indicating no evidence of poor fit. The model also showed moderate explanatory power, with a Nagelkerke R^2^ of 0.38. Overall model performance was significant (likelihood ratio test χ^2^ = 82.58, *p* < 0.001).

## Data Availability

The data supporting the findings of this study are not publicly available due to privacy considerations. Requests for access to the data should be directed to the corresponding author and will be considered on a case-by-case basis in accordance with institutional policies and applicable data protection legislation.

## Abbreviations

The following abbreviations are used in this manuscript:

DS	Down Syndrome
ACB	Anticholinergic burden
ICD-10	International Classification of Diseases-10
ATC	Anatomical Therapeutic Classification
